# Voxel Placement Precision for GABA-Edited Magnetic Resonance Spectroscopy

**DOI:** 10.4236/ojrad.2017.71004

**Published:** 2017-03-24

**Authors:** Xue Bai, Ashley D. Harris, Tao Gong, Nicolaas A. J. Puts, Guangbin Wang, Michael Schär, Peter B. Barker, Richard A. E. Edden

**Affiliations:** 1 Department of Radiology, Qilu Hospital of Shandong University, Jinan, China; 2 Russell H. Morgan Department of Radiology and Radiological Science, The Johns Hopkins University School of Medicine, Baltimore, MD, USA; 3 F. M. Kirby Research Center for Functional Brain Imaging, Kennedy Krieger Institute, Baltimore, MD, USA; 4 Department of MR, Shandong Medical Imaging Research Institute, Shandong University, Jinan, China

**Keywords:** MRS, Voxel Placement, Precision, Voxel Prescription, Registration

## Abstract

The purpose of the present study was to assess the reproducibility of voxel placement for GABA-edited MRS. GABA-edited MRS data were acquired in 13 healthy volunteers from (3 cm)^3^ voxel; and within the same session a second acquisition was independently prescribed. A three-dimensional voxel mask image was reconstructed in T1-image-space using the SVMask tool (in house software). Reproducibility of voxel placement was assessed using the Dice overlap coefficient, both within-subject and between-subject following co-registration of T1 images and transformation of voxel mask images to standard space. Within-subject overlap coefficients were 86% ± 5%. Between-subject overlap coefficients were 75% ± 10%. For the two voxel locations considered (occipital and sensorimotor), voxel overlap was very similar. Between-subject values are higher due to between-session effects, anatomical variability and volume mismatch in standard space. While surprisingly low in terms of volume overlap, the overlap coefficients correspond to acceptable linear displacements.

## 1. Introduction

1H magnetic resonance spectroscopy (MRS) is a non-invasive technique that allows for the quantitative investigation of a number of *in vivo* metabolites, such as N-acetyl aspartate, creatine and choline, commonly interpreted as markers for neuronal integrity, energetic status and membrane turnover respectively [1]. Recently, there has been significant interest in using edited MRS to reveal signals from less concentrated metabolites, such as GABA [2] [3], glutathione [4] and ascorbate [5]. Due to the fundamental insensitivity of MRS, larger measurement volumes are required for these edited experiments, typically on the order of (3 cm)^3^ compared to (2 cm)^3^ or less for traditional single-voxel MRS [6].

In cohort MRI studies, one common post-processing step is the co-registration of images either to each other, or to a standard-space template. Co-registration of images across a cohort allows comparisons between images to be drawn for anatomically equivalent regions, limited only by the quality of co-registration. In contrast, MRS is usually performed as a single-voxel measurement, and placement of voxels involves planning on the basis of predefined anatomical or functional landmarks. This is an irreversible anatomical judgment that cannot be mitigated by post-processing co-registration. For single-voxel acquisitions, the measurement acquired corresponds to the region prescribed without any further spatial resolution, or opportunity for spatial realignment. Conclusions drawn from MRS studies are usually based upon the functional role of a particular anatomical structure, and there is generally an implicit assumption that voxel placement is accurate and reproducible. To date, a landmark-based approach is the main method for edited MRS voxel placement. Given the reliance on anatomical landmarks, it is therefore particularly important to assess the reproducibility of voxel placement in MRS studies [7], especially for edited MRS experiments.

To our knowledge, no previous study has investigated the placement reliability of voxel for edited MRS, compared with traditional MRS. The increase in voxel size required for edited MRS of less concentrated metabolites may have several impacts on placement precision: displacement of the voxel by a fixed distance will have a relatively smaller impact on the voxel contents; larger voxels may suffer from additional anatomical restrictions; and operator care or judgment may be influenced by the size of the voxel to be planned. One further development that is relevant for studies of GABA is the increasing adoption of functionally motivated measurement regions, as opposed to anatomically defined studies. Tolerance of placement variance might be greater for a fronto-parietal region than a primary sensorimotor region, both because of the greater specificity of the functional definition and the qualitative difference in the conclusions likely to be drawn from such a study.

In this current study, the reproducibility of voxel placement for two anatomically defined and functionally motivated, regions of interest (occipital and sensorimotor), commonly used in MRS studies of sensory and motor function [8] [9] [10] [11], was assessed. Using the Dice overlap coefficient (DOC), placement precision both within-subject within-session, and between-subject was investigated. Differences between within- and between-subject results are discussed in terms of the interpretation of individually different anatomy and the limitations of the DOC.

## 2. Methods

### 2.1. Participants

13 healthy male subjects (all right handed, age 30 ± 6.1 years old) participated in the study. Only male participants were included to mitigate gender effects on brain anatomy and voxel localization. Written informed consent was obtained for each participant under the approval of the local Institutional Review Board prior to testing.

### 2.2. Edited-MRS

**Figure 1:**
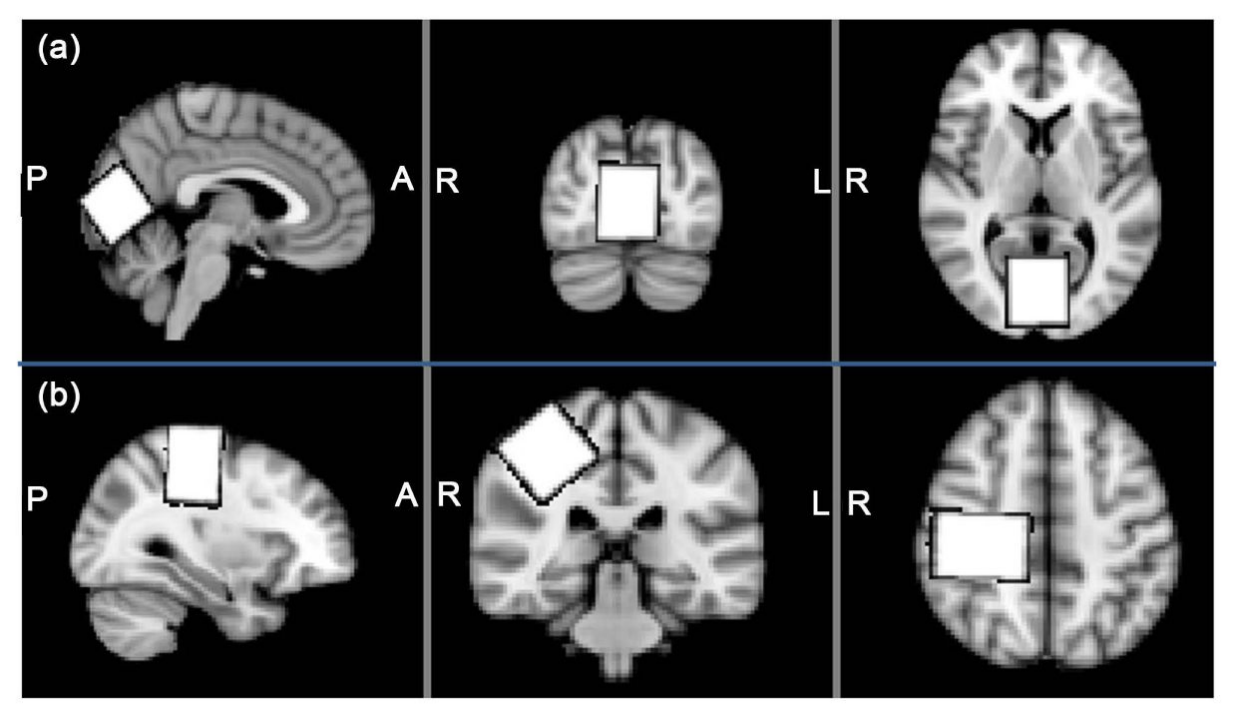
MRS voxel placement. A single-subject voxel, shown in white, is superimposed on the average template brain, in standard space. Both occipital (a) and sensorimotor (b) regions are considered.

Data were acquired on a Philips 3T “Achieva” MRI scanner (Best, the Netherlands) using a 32-channel head coil for receive and body coil for transmit. For each participant, sagittal 1 mm^3^ isotropic T1-weighted (T1w) images (MP-RAGE) were acquired and resliced in axial and coronal views (TR = 7.99 ms, TE = 3.76 ms, Flip angle = 8°). GABA-edited MRS voxels were manually placed in two regions (with the visualized voxel in Figure 1 correct for the 3 ppm GABA signal) viewing all 3 planes by a single experimenter. A (3 cm)^3^ voxel was placed on the right sensorimotor cortex (SM1, Figure 1(b)) and was centered on the central sulcus posterior to the hand-knob [12] in the axial plane; the voxel was rotated to align with the cortical surface by rotating in the coronal plane and subsequently in the sagittal plane. A second (3 cm)^3^ voxel was placed in the occipital cortex (OCC, Figure 1(c)), centered on the midline and rotated in the sagittal slice to align along the cerebellar tentorium and placed as posterior as possible without including the sagittal sinus or skull. Each voxel was placed twice in all participants. The first placement was part of a standard GABA-edited MEGA-PRESS scan with the following scan parameters: TE/TR = 68/2000 ms, 320 transients acquired with editing pulses placed at 1.9 (edit-ON) and 7.5 (edit-OFF) ppm, 2 k bandwidth and VAPOR water suppression(as described in [8]). The second placement was only performed to log voxel location parameters, although minimal MRS data were acquired (a 12-second water acquisition). Prior to the second placement, voxel location and angulations were zeroed so that the voxel was centered approximately in the center of the brain without rotation, and independent placement was again performed on the basis of the landmarks described above. All voxel placements were performed by a single experimenter and participants were not removed from the scanner in between the first and second voxel placement. No additional information (e.g. screen shots of the first placement) was used for the second placement. A total of 51 MRS voxels’ data, from 13 participants were included in this study (1 subject’s second SM1 voxel was unavailable).

### 2.3. Analysis

**Figure 2:**
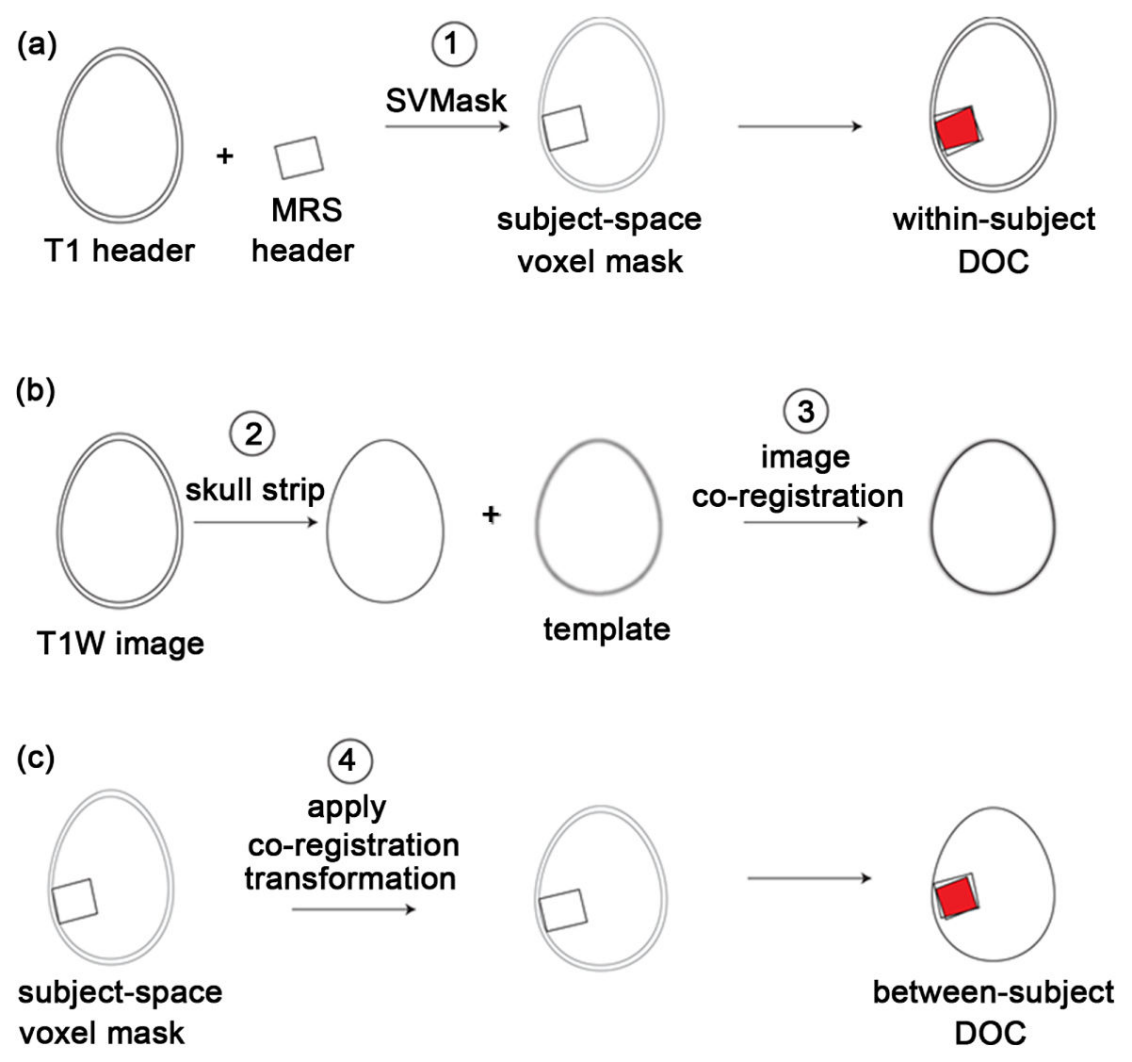
Schematic outline of the registration framework. Numbered steps correspond to the list in Analysis Methods above. (a) Voxel header information is used to reconstruct a voxel mask image (step 1). Masks from the two voxel placements (per region) are then compared to give the within-subject DOC; (b) T1W images are skull stripped (step 2) and co-registered to a standard-space template (step 3); (c) The co-registration transformation is applied to voxel masks (step 4), to allow between-subject DOC comparisons.

The following image analysis pipeline was used (Figure 2):

1. Generation of the MRS voxel mask (Figure 2(a)). Each MRS acquisition volume was reconstructed as a binary mask in the image matrix of the T1w image of the same subject using the SVMask tool (in house software), which extracts the required geometric information from MRS and MRI file headers.
2. Brain extraction (Figure 2(b)). Skull-stripping of 3D T1w images was performed using the Brain Extraction Tool (BET, v2.1) [13], from the FSL suite.
3. Image co-registration (Figure 2(b)). T1w images were co-registered to (2 mm)^3^ MNI standard-space brain using FMRIB’s Linear Image Registration Tool (FLIRT, v6.0) [14].
4. Voxel transformation to standard space (Figure 2(c)). For each subject, the transformation matrix determined in step 3 was applied to all the voxel masks generated in step 1 to give voxel masks in standard space (as shown in Figure 1).

#### 2.3.1. Within-Subject Overlap

The quantification of voxel overlap within subjects between the two scans was performed using the Dice overlap coefficient (DOC [15]). The DOC is defined as the intersection volume, divided by the mean volume of the two voxels; it ranges between 0 and 1, where 1 represents perfect overlap. For example, A might refer to the first OCC voxel mask and B to the second OCC voxel mask:

$$\text{DOC}=\frac{2\left(A\cap B\right)}{A+B}.$$

Within-subject voxel overlap DOC was calculated using FSL tools in subject-space (rather than standard space), prior to step 2 above (as shown in Figure 2(a)).

#### 2.3.2. Between-Subject Overlap in Standard Space

Between-subject voxel placement reliability was calculated using the first-placement voxel masks for each region from the 13 participants, registered to standard space. For each of the thirteen subjects, another subject was selected randomly (without replacement and prohibiting double comparisons e.g. 1–8 and 8-1) to generate thirteen unique pair-wise comparisons. The DOC was calculated for OCC and SM for each of these pairs. This process was repeated five times, so that in all 65 between-subject overlap coefficients were calculated for each region.

#### 2.3.3. Voxel Density Images

In standard space, an image was calculated of the sum of the voxel masks of all subjects (separately for OCC and SM voxel). For each point in space, this image reflects how often that point is included in the different subjects’ MRS voxels.

## 3. Results

### 3.1. Within-Subject Voxel Overlap

**Figure 3:**
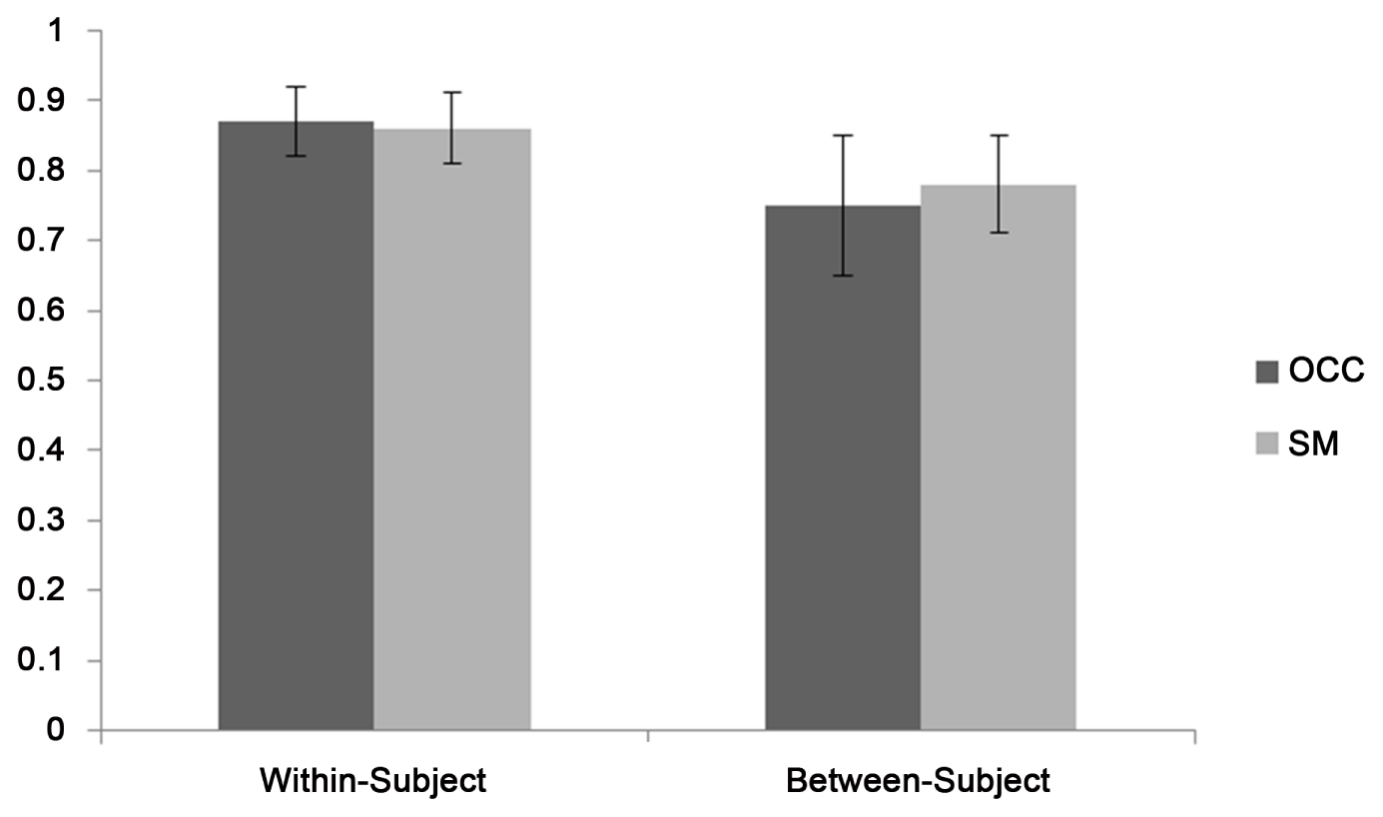
Overlap coefficients (DOC) calculations of MRS voxels within- and between-subjects. OCC = occipital; SM = sensorimotor.

The overlap between the first and second voxel prescriptions for each subject were 87% ± 5% in the occipital region and 86% ± 5% in the SM region (Figure 3). The displacement between the centers of the two voxels was 2.6 ± 1.2 mm (mean ± standard deviation), with very similar average displacements for both the OCC and SM locations (2.66 mm for SM and 2.63 mm for OCC).

### 3.2. Between-Subject Voxel Overlap

**Figure 4:**
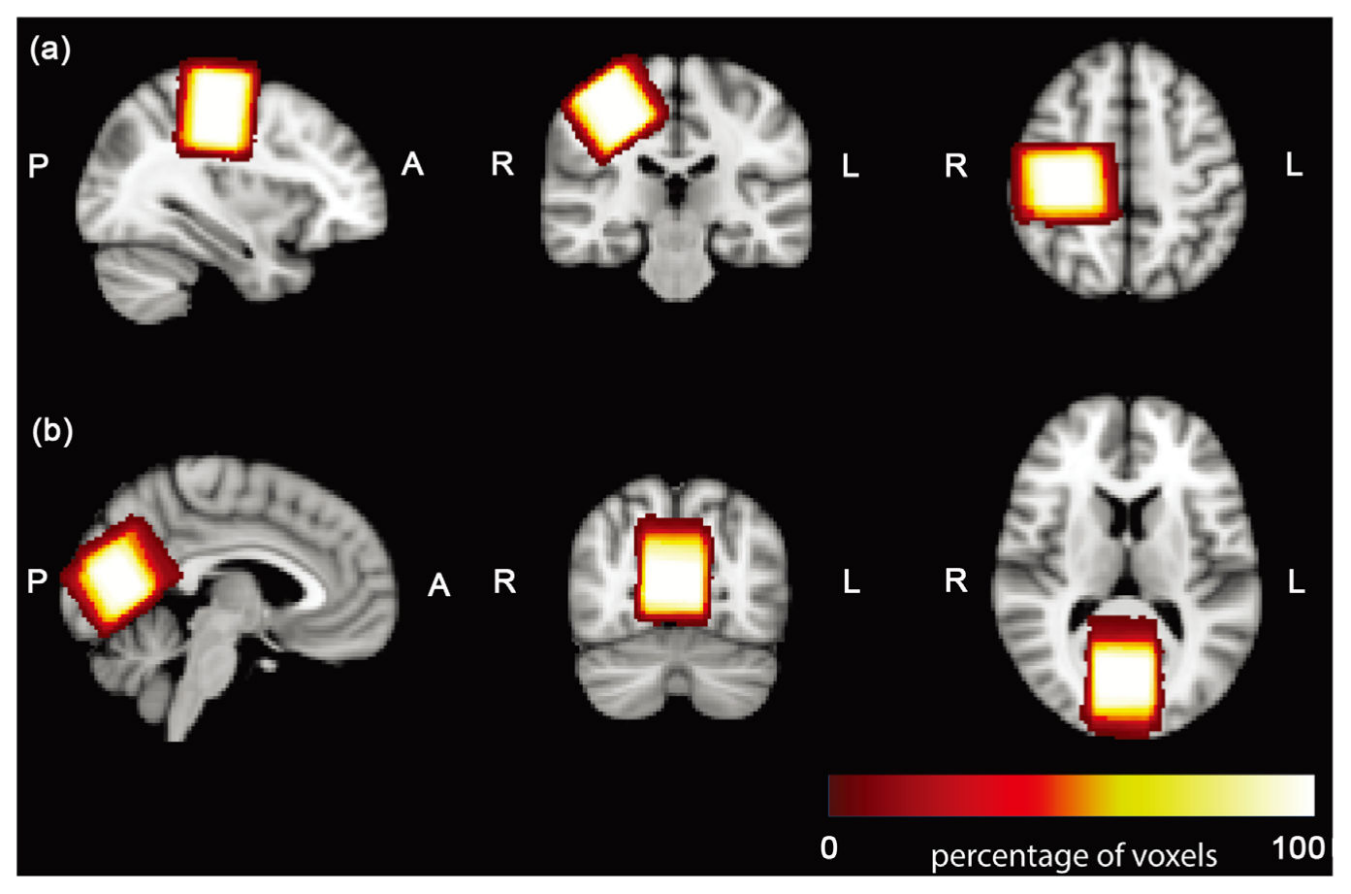
Voxel density images. All the OCC (a) and SM (b) voxels are transferred to one standard space. The degree of spatial overlap illustrates the repeatability of MRS voxel placement procedures.

Mean between-subject voxel overlap was 75% ± 10% in OCC and 78% ± 7% in SM (as shown in Figure 3 and Figure 4). Due to substantial variation in brain volume between subjects (from 1.05 liter to 1.50 liter for males [16]), the volume of the MRS voxels is scaled in standard space through the registration process. Voxel volumes were scaled relative to the mean by −13% to +15% (standard deviation 9%). The mean pair-wise volume mismatch is 11%.

## 4. Discussion

It is a tacit assumption of the majority of single-voxel MRS studies that metabolite concentrations are measured from equivalent regions in each subject. It is therefore somewhat surprising that the fidelity of voxel placement has only occasionally been investigated in the literature [7] [17]. In order to evaluate the repeatability of voxel placement for MEGA-PRESS GABA scans, this study calculated the DOC both within-subject and, after image co-registration, between-subject. The primary results suggest the within-subject DOC is 85% in both occipital and sensorimotor measurement regions, and that the between subject DOC is 75% for a 3 × 3 × 3 cm^3^ voxel.

At first glance, these overlap numbers are surprisingly low; however, 85% overlap is equivalent to 5% (or 1.5 mm of one direction of a (3 cm)^3^ voxel) displacements along each of the three spatial directions, without any variability in rotations. The mean displacement between voxel centers is 2.6 mm, equal to the diagonal of a 1.5 mm cube, suggesting that displacement accounts for the majority of the overlap loss, with only minor losses due to rotation. Given that voxels are placed using T1w images with 1-mm-isotropic resolution, precision better than 1 mm in each direction would not be expected. Similarly, the 75% overlap between subjects corresponds to a 9% (or 2.7 mm) displacement along all three axes-again not substantially greater than the (2 mm)^3^ matrix on which co-registration was performed.

Some limitations arise from the choice of the Dice coefficient (DOC). Firstly, the DOC only reports on the overlap between the tissues contained in different voxels, and cannot address the impact that any change in voxel contents has on measured GABA concentration. Secondly, it is difficult to interpret the difference in DOC from within-to between-subjects comparisons (85% vs. 75%). Some of this reduction in overlap is “real”, reflecting the operator’s variable interpretation of individual anatomy, while some of it is artifactual reflecting imperfections in co-registration on a (2 mm)^3^ matrix.

A further limitation of using the Dice coefficient in standard space is that two voxels with identical position and orientation that originate from different-sized brains will not give overlap of 100% due to a volume mismatch in standard space. In this special case, the Dice coefficient is less than 1 by half the fractional difference in volume between the brains. This suggests that the mean volume mismatch in our cohort (11%) therefore accounts for about half of the additional between-subject overlap loss compared to within-subject. Thus, while the DOC reflects the mathematical overlap between voxels, in standard space it does not report simply on operator reliability. One might even suggest that voxel volume should be scaled relative to total brain volume when MRS scans are prescribed (which would likely result in increased DOC), but this has signal-to-noise (SNR) and data quality implications also. Additional limitations are the consideration of only two voxel positions, the lack of within-subject between-session data, and the single operator prescribing voxels. Although the strong agreement between the two regions studied suggests that the findings may be generalizable, these results are likely to be affected to some degree by several factors including the complexity of the prescription protocol including the number of rotations, reproducibility of subject placement in the scanner (*i.e.* brain orientation in the anatomical images), and ease of identification of landmarks used (which may differ due to lesions, atrophy or normal/abnormal anatomical variation). Additional advances in co-registration, as well as an in-depth investigation of the effect of small changes in voxel tissue composition on GABA levels, would allow for a better understanding of the effect of small changes in voxel placement within and between subjects. Furthermore, we restricted our investigation to single-prescriber, single-scanner and single-session. For longitudinal or multi-center studies, the effects of multi-prescriber, multi-scanner, and multi-session on voxel localization and overlap would need to be investigated as well.

In practical terms, this study shows that voxel placement to a precision of 2 – 3 mm in three directions is possible, with care. Although these results give surprisingly low Dice coefficients of ~75%, this level of precision is approximately equal to the ability of subjects to remain motionless during the 10-minute scan. This agreement is only possible due to the rigorous specification of voxel placement protocol, including three-dimensional position and rotation information. Of particular note is the need to specify the order of multiple voxel rotations (which do not commute) for voxels. A range of voxel overlaps have been shown previously, from 57% within-subject for a small (15 mm)^3^ parietal voxel [7] to 86% for a slightly larger (20 mm)^3^ posterior cingulate voxel [17]. There is some evidence that automated voxel placement protocols, which typically calculate voxel location parameters from parameters co-registering images to standard space within-session, can perform as well as human operators and remove some variance [17], and prospective voxel placement correction has been demonstrated with a navigator-based acquisition [18]. Another methodological improvement is the use of voxel density maps (as used in Gaetz *et al*. [19]) to simultaneously display the position of MRS voxels and the placement precision across subjects.

## 5. Conclusion

In conclusion, the percentage of equivalent tissue included by 3 × 3 × 3 cm^3^ MRS voxels in different subjects is surprisingly low at 75%. However, this corresponds to displacements of less than 3 mm along three axes, which seems to be relatively good agreement, and some fraction of this overlap loss is caused by brain volume mismatches. Within-subject agreement is ~85%, again low at first glance, but equivalent to acceptable 3D displacements of 1.5 mm in all three directions.
